# Prophylactic use of lamivudine for hepatitis B exacerbation in post-operative breast cancer patients receiving anthracycline-based adjuvant chemotherapy

**DOI:** 10.1038/bjc.2011.4

**Published:** 2011-02-01

**Authors:** J Yun, K H Kim, E S Kang, G-Y Gwak, M S Choi, J E Lee, S J Nam, J-H Yang, Y H Park, J S Ahn, Y-H Im

**Affiliations:** 1 Division of Hematology-Oncology, Department of Medicine, Samsung Medical Center, Sungkyunkwan University School of Medicine, 50 Irwon-dong Gangnam-gu, Seoul 135-710, Korea; 2 Department of Laboratory Medicine, Samsung Medical Center, Sungkyunkwan University School of Medicine, 50 Irwon-dong Gangnam-gu, Seoul 135-710, Korea; 3 Department of Medicine and Digestive Disease Research Centre, Samsung Medical Center, Sungkyunkwan University School of Medicine, 50 Irwon-dong Gangnam-gu, Seoul 135-710, Korea; 4 Department of Surgery, Samsung Medical Center, Sungkyunkwan University School of Medicine, 50 Irwon-dong Gangnam-gu, Seoul 135-710, Korea

**Keywords:** hepatitis B reactivation, breast cancer, doxorubicin, adjuvant chemotherapy

## Abstract

**Background:**

With the increasing incidence of breast cancer worldwide, in particular in southeast Asia (including Korea), and the common use of anthracyclines in the adjuvant and metastatic settings, the occurrence of Hepatitis B virus (HBV) reactivation may develop in this patient population. The use of prophylactic antiviral agents in cancer patients may result in a reduced HBV exacerbation. The purpose of the current study was to assess the efficacy of prophylactic lamivudine in reducing the incidence and severity of HBV reactivation in post-operative breast cancer patients undergoing adjuvant doxorubicin-containing chemotherapy.

**Methods:**

The medical records of patients undergoing anthracycline-based adjuvant chemotherapy at Samsung Medical Center between January 2001 and September 2008 were reviewed.

**Results:**

From the database, 1912 breast cancer patients who had received anthracycline-based adjuvant chemotherapy were identified. Of 131 patients who were HBV surface antigen positive, 55 and 76 did and did not receive prophylactic lamivudine, respectively. In all, 30 patients (23%) developed hepatitis during doxorubicin-containing adjuvant chemotherapy. Of the 30 patients, 5 (9%) were in the prophylactic lamivudine group and 25 (33%) in the control group (*P*=0.001). In the prophylactic lamivudine group, there was significantly less HBV reactivation (1 patient (2%) *vs* 20 patients (16%); *P*=0.002), less disruption of chemotherapy (7 *vs* 14% *P*=0.04), and less severe hepatitis (0 *vs* 17% *P*=0.002).

**Conclusion:**

Prophylactic lamivudine significantly reduced the incidence and severity of HBV reactivation in breast cancer patients undergoing anthracycline-based adjuvant chemotherapy.

Hepatitis B virus (HBV) infection is a global health problem and particularly endemic in southeast Asia and the western Pacific regions, where >8% of the population are chronic HBV carriers (Lok *et al*, 1987; Lee, 1997). The prevalence of HBV surface antigen (HbsAg) in Korea is 5.1% in men and 4.1% in women according to the National Health and Nutrition Survey (Lee *et al*, 2002).

Reactivation of HBV replication with an increase in serum HBV DNA and alanine aminotransferase (ALT) activity has been reported in 20–50% of hepatitis B carriers undergoing cytotoxic chemotherapy for cancer treatment (Yeo *et al*, 2000, 2003, 2004b; Idilman *et al*, 2004; Lok and McMahon, 2007). During intense cytotoxic therapy, immunosuppression may allow enhanced HBV replication, which results in a widespread infection of hepatocytes. With the subsequent restoration of immune function due to the withdrawal of cytotoxic or immunosuppressive therapy, there is a rapid immune-mediated destruction of HBV-infected hepatocytes, which is manifested clinically as asymptomatic self-limiting hepatitis, severe progressive hepatic failure, and even death (Lau *et al*, 2003; Mindikoglu *et al*, 2006). The prognosis of cancer may be compromised by disruption in anticancer treatment during the course of hepatitis, with a delay in treatment cycles and premature termination of chemotherapy (Lok *et al*, 1991).

HBV reactivation has been reported frequently in patients diagnosed to have lymphoma or breast cancer and those who have received anticancer chemotherapy, and in this setting, the use of anthracyclines and corticosteroids as part of the chemotherapeutic combination and/or anti-emetic pre-medication were the factors shown to be associated with HBV reactivation (Khokhar *et al*, 2009). The use of antiviral agents in cancer patients has been shown to reduce HBV reactivation and to prevent the associated fatal complications. Consensus guidelines by the American Association for the Study of Liver Diseases (AASLD) recommend the use of prophylactic antiviral agents for HBsAg-positive cancer patients undergoing cytotoxic chemotherapy (Lok and McMahon, 2007).

Lamivudine, a cyclic nucleoside analogue, is effective in suppressing HBV DNA, normalising liver enzymes, and improving the histological features in HBeAg-positive and -negative/HBV DNA-positive patients. This effect suggests that prophylactic antiviral therapy with lamivudine reduces the risk of HBV reactivation and prevents the associated fatal hepatic complications with no myelosuppressive effect in patients already exposed to an immunosuppressive and cytotoxic treatment. The use of prophylactic lamivudine in cancer patients undergoing chemotherapy has recently been reported in several small series, which were based mainly on patients with lymphomas or a heterogeneous group of various cancers treated with steroids and rituximab. The findings of the studies suggested that the antiviral agent, lamivudine, may reduce HBV reactivation and the associated morbidity (Lok *et al*, 1991; Lau *et al*, 2003; Yeo *et al*, 2004b; Mindikoglu *et al*, 2006). Furthermore, according to a recent study involving patients in Hong Kong, prophylactic lamivudine treatment in post-operative and metastatic breast cancers significantly reduced the incidence of HBV reactivation from 31 to 7%, with a decrease in hepatitis from all causes from 59 to 13%. In addition, disruption in chemotherapy was significantly reduced from 46 to 16% (Yeo *et al*, 2004b). However, the population also included patients with hepatic metastasis, thus the results should be interpreted with caution. Furthermore, many studies involving prophylactic antiviral therapy have been limited by small sample size, heterogeneity in tumour type, heterogeneous disease settings (adjuvant or palliative treatment), and varying chemotherapy regimens, and it is difficult to determine how effective lamivudine is in breast cancer patients receiving adjuvant chemotherapy, especially in patients treated with a doxorubicin-containing regimen, the most commonly used and effective agent in adjuvant treatment.

The objective of the present study was to assess the efficacy of prophylactic lamivudine in reducing the incidence and severity of HBV reactivation in post-operative breast cancer patients undergoing adjuvant doxorubicin-containing chemotherapy.

## Patients and methods

At the time of surgery, liver function tests, sonography of the liver, and serological examinations for hepatitis B (HBsAg, anti-HBs, and anti-HBc IgG) were performed as a screening procedure. For the duration of adjuvant chemotherapy, complete blood cell counts and liver function tests were performed before each cycle. When exacerbation of hepatitis B was documented, appropriate management for the treatment of hepatitis was followed. Planned chemotherapies were modified (doses and schedules) according to the severity of hepatitis. The 131 patients were divided into two groups (patients who did (*n*=55) and did not (*n*=76) receive prophylactic lamivudine). Most of the HBsAg-positive patients in our Institute were treated with lamivudine as a pre-emptive antiviral therapy since 2005 (post-lamivudine era) compared with 2005 (pre-lamivudine era). We analysed the clinical manifestations with the laboratory findings of hepatitis during and after adjuvant chemotherapy. Our study protocol was approved by the Institutional Review Board of Samsung Medical Center.

### Hepatitis serology and HBV DNA assay

Hepatitis B was detected by commercial enzyme immunoassay (HBsAg; Abbott, Chicago, IL, USA). The status of hepatitis B e-antigen (HBeAg, AxSYM; Abbott) and HBV DNA were evaluated in HBsAg-positive patients. Serum HBV DNA levels were assessed using the Digene Hybrid Capture II HBV DNA test (Digene Corp., Gaithersburg, MD, USA) with a limit of detection of 0.5–6000 pg ml^−1^.

### Definitions

The following definitions according to Lok and McMahon (2007) and subsequently modified by Lok and McMahon (2009) were applied. ‘Hepatitis’ was defined as a ⩾3 times increase in ALT that exceeds the upper normal limit (UNL, <40 IU l^−1^) or an absolute increase of ALT to >100 IU l^−1^ when compared with the baseline pre-chemotherapy value. Hepatitis, attributable to ‘HBV reactivation,’ was defined as an increase in the HBV DNA level ⩾10 times when compared with the baseline level or an absolute increase in the HBV DNA level that exceeded 1000 × 10^6^ g.E. ml^−1^ in the absence of other systemic infections. The severity of hepatitis was defined as ‘mild,’ ‘moderate,’ and ‘severe’ when the rise in ALT was ⩽2 × the UNL, >2 × and ⩽5 × the UNL, and >5 × the UNL, respectively. ‘A disruption in chemotherapy’ was defined as a premature termination of chemotherapy or a delay of ⩾8 days of chemotherapy between cycles.

### Adjuvant chemotherapy and use of lamivudine

The anthracycline-based chemotherapy regimens included AC (doxorubicin plus cyclophosphamide), FAC (5-FU plus doxorubicin plus cyclophosphamide), and AC followed by four cycles of a taxane (paclitaxel (175 mg m^−2^) or docetaxel (75 mg m^−2^)). The AC chemotherapy consisted of doxorubicin (60 mg m^−2^) and cyclophosphamide (600 mg m^−2^) i.v. push on day 1 for four cycles. The FAC consisted of cyclophosphamide (500 mg m^−2^), doxorubicin (50 mg m^−2^), and 5-FU (500 mg m^−2^) i.v. push on day 1 for six cycles. The chemotherapy cycles were repeated every 21 days. Patients with primary tumours ⩾5 cm in size or ⩾4 positive nodes, and patients who underwent breast-conserving surgery were treated with adjuvant radiotherapy after chemotherapy. Patients with ER- or PR-positive tumours were treated for 5 years with tamoxifen or aromatase inhibitor therapy after adjuvant chemotherapy, according to the patient's menopausal status.

Patients in the prophylactic lamivudine group commenced lamivudine (100 mg orally daily) within 7 days before the start of chemotherapy. For patients with renal insufficiency, the daily lamivudine dose was adjusted according to creatinine clearance. In the control group, prophylactic lamivudine was not administered. All of the patients received lamivudine after HBV reactivation.

### Statistical analysis

Clinical and laboratory variables were compared across groups using the Fisher's exact test or Pearson's *χ*^2^ test where appropriate for dichotomous variables, and the Mann–Whitney *U*-test for continuous variables. A *P*-value <0.05 was considered statistically significant. The Cox proportional hazards regression model was used in univariate and multivariate analyses to identify the most significant prognostic factors for HBV reactivation. The logistic regression model was created using an unconditional procedure, and the probability was set at 0.05 for entry and 0.10 for removal. The hazard ratio (HR) and 95% confidence interval (CI) were estimated with a pre-determined reference risk of 1.0. SPSS software (version 17.0; SPSS Inc., Chicago, IL, USA) was used for statistical analyses.

## Results

### Patient characteristics

**Figure 1:**
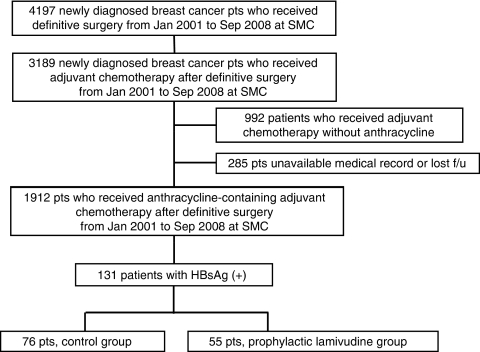
Patients' cohort. F/u, follow up.

Among 3189 newly diagnosed breast cancer patients who underwent curative surgery between January 2001 and September 2008 at Samsung Medical Center, 1912 receiving anthracycline-based adjuvant chemotherapy were identified. All 3189 patients underwent HBsAg testing. In Korea, all patients who received elective surgery routinely underwent HBsAg testing (at least HBsAg/sAb) before surgery. Of the 1912 patients, 131 HBsAg-positive patients (6.9%) with normal hepatic function (serum levels of aspartate aminotransferase (AST), ALT, alkaline phosphatase, albumin, and total and direct bilirubin) were identified (Figure 1). None of the patients had a previously received anti-HBV therapy. In all, 55 and 75 patients did and did not receive prophylactic lamivudine, respectively. The patient's characteristics are provided in Table 1. There were no significant differences with respect to age, level of ALT, total bilirubin, and adjuvant radiation therapy. The baseline AST level had a tendency to be higher and the albumin level had a tendency to be lower in the prophylactic lamivudine group (Table 1).

### Biochemical and clinical outcomes in the two groups of patients

#### The prophylactic lamivudine group

Patients received lamivudine (100 mg daily) for a median duration of 185 days (range, 30–1540 days). Treatment was continued for a median of 2 months (range, 0–47 months) after completion of chemotherapy. The median number of chemotherapy cycles was 6 (range, 4–8 cycles). Two patients developed a tyrosine–methionine–aspartate–aspartate (YMDD) mutation during lamivudine therapy, and therefore received adefovir dipivoxil (10 mg) add-on to lamivudine. The total duration of lamivudine therapy in these two patients was 1540 days and 745 days, respectively.

Five patients (9%) developed hepatitis during or after chemotherapy and one patient (2%) developed HBV reactivation 15 days after cessation of lamivudine (Table 2). The severity of hepatitis was mild in three patients and moderate in two patients. In one case of HBV reactivation, the severity of hepatitis was moderate. The median duration from initiation of chemotherapy to development of hepatitis was 68 days (range, 44–351 days).

Disruption in chemotherapy occurred in two patients (3%). There were no mortalities among patients who received prophylactic lamivudine. The antiviral agents were well tolerated and were not associated with any unexpected or additional toxicities to chemotherapy.

#### The control group

The median number of chemotherapy cycles was six (range, 1–9 cycles). In all, 25 of the 76 control group patients (33%) developed hepatitis during or after chemotherapy. Among the 75 patients, 16 (21%) had HBV reactivation. The severity of hepatitis was mild in 3, moderate in 7, and severe in 15 patients. In patients with HBV reactivation, 3 had mild hepatitis, 7 had moderate hepatitis, and 15 had severe hepatitis. One patient died because of rapid progression of recurrent breast cancer during third-line palliative chemotherapy, which was not related to hepatitis B reactivation. Disruption in chemotherapy occurred in 11 patients. Two patients had premature termination of chemotherapy, and nine patients completed chemotherapy with interval delays (Table 2).

Of the 16 patients who developed HBV reactivation, 14 received lamivudine and 2 received entecarvir as a therapeutic measure at the time of HBV reactivation. Despite this, 11 patients (14%) had disruptions in chemotherapy, 2 had premature termination of chemotherapy, and 9 had prolonged interval delays.

#### Comparison of outcomes between the two groups

In the prophylactic lamivudine group, there was significantly less HBV reactivation (2 *vs* 21% in the controls, *P*=0.001), a lower incidence of hepatitis (9 *vs* 33%, *P*=0.001), and less disruption in chemotherapy (3 *vs* 14%, *P*=0.04).

#### Prognostic factors for HBV reactivation

Univariate analysis showed a lower rate of HBV reactivation in patients who received prophylactic lamivudine than in those who did not (21 *vs* 2%, respectively; *P*=0.001). There was no independent prognostic factor for HBV reactivation other than prophylactic lamivudine. Multivariate analysis showed that ‘no use of prophylactic lamivudine’ was a significant risk factor (HR, 14.6; 95% CI, 1.89–114.13; *P*=0.031; Table 3).

## Discussion

In the current study, we confirmed the importance of prophylactic lamivudine treatment in HBV carriers receiving adjuvant anthracycline-based chemotherapy for post-operative early breast cancer. Considering that anthracyclines are some of the most widely used and effective drugs to treat patients with breast cancer in the adjuvant setting as well as patients with metastatic disease (Bennett *et al*, 1988; Henderson *et al*, 1989; Green *et al*, 1996; Fossati *et al*, 1998; Pai and Nahata, 2000; Crown *et al*, 2002), the number of reports is limited (Lai *et al*, 1998; Rossi *et al*, 2001; Lee *et al*, 2003). These results included a heterogeneous population in terms of the use of a prophylactic antiviral agent. Indeed, this is the first study involving the role of antiviral prevention in breast cancer patients receiving anthracycline-based adjuvant chemotherapy.

Pre-emptive antiviral therapy has been used as a potential benefit of antiviral agents for HBV as lamivudine had been known, although still there are lacking data to support the pre-emptive therapy for this clinical setting. Actually, most of the HBsAg-positive patients have been treated with lamivudine as pre-emptive antiviral therapy after 2005 in our Institute (post-lamivudine era). Before 2005, we did not use lamivudine as pre-emptive antiviral therapy (pre-lamivudine era). Thus, this clinical decision was made not by each physician's preference, but by the ‘changing paradigm’.

Using a prophylactic lamivudine significantly reduced the incidence of hepatitis from 33 to 9%, HBV reactivation from 21 to 2%, and disruption of chemotherapy from 14 to 3% in HBV-carrier breast cancer patients who are undergoing anthracycline-based adjuvant chemotherapy. The implication of this study focused on breast cancer patients receiving anthracyline-containing adjuvant chemotherapies. Thus, morbidity and mortality caused by adjuvant treatment should not override cancer outcomes.

In contrast, in the control group, although all 16 patients who developed HBV reactivation received therapeutic antiviral treatment, 2 of the 16 patients (13%) had premature termination of chemotherapy. Therefore, prophylactic antiviral treatment is more important than therapeutic interventions after viral reactivation with respect to cancer outcome in HBV carriers undergoing anthracycline-based chemotherapy.

Selection of the population of patients receiving adjuvant chemotherapy can reduce bias, which may be underestimated or overestimated hepatitis itself associated with hepatic metastasis. In addition, assuming the popularity of doxorubicin and cyclophosphamide in this common homogeneous clinical setting, the role of lamivudine prophylaxis appeared to be effective in immunosuppressive therapies. This finding is supported by the data, in which the use of anthracycline-contating chemotherapy is identified as a risk factor in reactivation of hepatitis B (Yeo *et al*, 2004a). Although there were no mortalities in either group, severe hepatitis occurred in 15 patients (13%) in the control group, suggesting the possibility that mortality would occur. We should thus pay attention to the possibility of mortality related to HBV reactivation during adjuvant chemotherapy in this population of curable patients. As level I evidence, the AASLD guidelines recommend that lamivudine can be used even if the anticipated duration of treatment is short (⩽12 months) and serum HBV DNA is not detectable at baseline. Tenofovir or entecavir is preferred if a longer duration of treatment is anticipated (level III evidence). Thus far, most of the studies have focused on the effect of prophylactic lamivudine treatment regardless of the anticipated duration of the treatment. Therefore, we also selected an adjuvant setting to precisely assess the efficacy of prophylactic lamivudine according to the recommendations.

The AASLD guidelines recommend beginning antiviral therapy 7 days before chemotherapy and continuing for 6 months (level III evidence; Yeo *et al*, 2004a, 2004b). The duration of treatment has not been established. In the case of chronic HBV infection, prolonged lamivudine therapy exceeding 6 months in duration has been associated with an increased likelihood of treatment-emergent HBV variants with a YMDD mutation, appearing in 12–20% in the first year of treatment (Lau *et al*, 2003; Yeo *et al*, 2004b). In the current study, the median duration of lamivudine treatment was 185 days, and 30 of 55 patients received prolonged lamivudine treatment exceeding 6 months. Among 30 patients, a YMDD mutation emerged in two patients (7%). One case occurred in the course of using lamivudine for 24 months and the other case occurred in the course of using lamivudine for 51 months. Since then, the patients have received combination treatment with lamivudine and adefovir. Fortunately, there were no occurrences of HBV reactivation since that time. Accordingly, despite the potential benefits of the prophylactic approach, careful clinical monitoring is still required. Furthermore, there is a need to emphasise the implication, especially in patients in the adjuvant treatment setting. We must consider the variability of the prevalence of hepatitis B, even though a recent ASCO guideline update does not recommend prophylaxis universally (Artz *et al*, 2010). Although the prevalence of chronic HBV infection is decreasing, it is still a major aetiology of liver cirrhosis and hepatocellular carcinoma in Korea (Chae *et al*, 2009). Hepatitis B reactivation can be a cause of major morbidity and mortality in patients who receive chemotherapy in this endemic area. Thus, we need to consider the area to adapt the recommendations universally. In addition, large prospective randomised trials are needed to confirm the value of pre-emptive therapy in this setting.

We confirmed the efficacy of prophylactic lamivudine with respect to reducing the incidence and severity of HBV reactivation in patients undergoing adjuvant anthracycline-based chemotherapy in a homogeneous group of breast cancer patients. We fully expect that updated cancer treatment guidelines will emerge soon, including guidelines of prophylactic antiviral treatment for each cancer, because suggesting the need for prophylactic antiviral treatment for each cancer is a significant task.

## Figures and Tables

**Table 1:** Comparison of clinical characteristics between the ‘control’ and the ‘prophylactic lamivudine’ groups in the HBsAg-positive breast cancer patients

	**Control group**		**Prophylactic lamivudine group**		
	***N***	**%**	***N***	**%**	***P*-value**
No. of patients	76	58	55	42	
					
Age (years) median, range	46 (30–69)		48 (30–68)		0.18
					
*Pre-chemotherapy status*					
AST (IU l^−1^) (0–40)^a^, mean	24		30		0.09
ALT (IU l^−1^) (0–40)^a^, mean	25		25		0.25
Total bilirubin (mg dl^−1^) (0.2–1.5)^a^, mean	0.5		0.6		0.11
Albumin (g dl^−1^) (3.5–5.2)^a^, mean	4.2		4.0		0.06
					
*Chemotherapy regimen*					
AC	17	22	3	5	
FAC	28	37	25	45	0.02
AC → D/T	31	41	27	50	
					
Steroid use for anti-emetics	33	45	28	51	0.48
					
*Stage*					
1	11	15	9	16	
2	39	51	32	58	0.56
3	26	34	14	26	
					
Adjuvant RT	12	16	4	7	0.18

Abbreviations: AC=doxorubicin + cyclophosphamide; AC → D/T=AC followed by docetaxel or paclitaxel; ALT=alanine-aminotransferase; AST=aspartate aminotransferase; FAC=5-FU + doxorubicin + cyclophosphamide; HbsAg=hepatitis B virus surface antigen; RT=radiation therapy.

a Normal range.

**Table 2:** Morbidity and mortality in the ‘control’ and the ‘prophylactic lamivudine’ groups

	**Control group**		**Prophylactic lamivudine group**		
	***N***	**%**	***N***	**%**	***P-*value**
No. of patients	76		55		
					
*Overall morbidity*					
Incidences of hepatitis	25	33	5	9	0.001
Hepatitis attributable to HBV reactivation	16	21	1	2	0.001
*Severity of hepatitis*					0.001
Mild (HBV reactivation)	3 (2)	4	3 (0)	7	
Moderate (HBV reactivation)	7 (1)	10	2 (1)	3	
Severe (HBV reactivation)	15 (13)	19	0 (0)	0	
HBV DNA at reactivation (mean, pg)	128 970		93 567		0.132
					
*Disruptions of chemotherapy*	11	14	2	3	0.04
*Premature termination*	2	3	0	0	
Hepatitis due to HBV reactivation	2	3	0	0	
*Completion of chemotherapy with delay of* >*8 days*	9	12	2	3	
Hepatitis, all cases	9	12	2	3	
Hepatitis due to HBV reactivation	5	7	0	0	
Other causes	0	0	0	0	
					
*Overall mortality related to hepatitis B reactivation*	0	0	0	0	
Median no. of chemotherapy cycles received (range)	6.1 (1–9)		6.8 (4–8)		0.018

Abbreviation: HBV=Hepatitis B virus.

**Table 3:** Multivariate logistic-regression analysis on reactivation of hepatitis B

	***P*-value**	**HR**	**95% CI**	
No use of prophylactic lamivudine	0.031	14.6	1.89	114.13
Radiation	0.891	1.4	0.80	4.52
Stage	0.752	1.6	0.02	8.23

Abbreviations: CI=confidence interval; HR=hazard ratio.
